# Supplementary material for: Interface-mediated hygroelectric generator with an output voltage approaching 1.5 volts
Source: Nat Commun. 2018 Oct 9;9:4166. doi: 10.1038/s41467-018-06633-z (PMC6177432; doi:10.1038/s41467-018-06633-z)
Supplement: Supplementary file 1 — Supplementary information [file 41467_2018_6633_MOESM1_ESM.pdf]

Supplementary Information

Interface-mediated hygroelectric generator with an output voltage approaching to 1.5 V

Huang et al.

Supplementary Figures

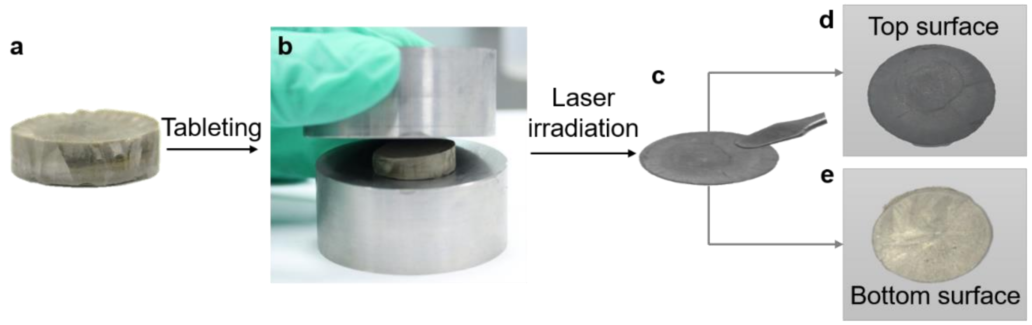

Supplementary Figure 1. Fabrication process of heterogenous graphene oxide. (a) Graphene oxide (GO) foam and, (b) the subsequent tableting treatment process under 10 KN for 10 min. (c) Directionally-controlled reduction of GO membrane with a 450 nm laser (15 W). (d, e) The top surface and bottom surface of the heterogeneous membrane at c. The diameter of the sample foam is 2 cm.

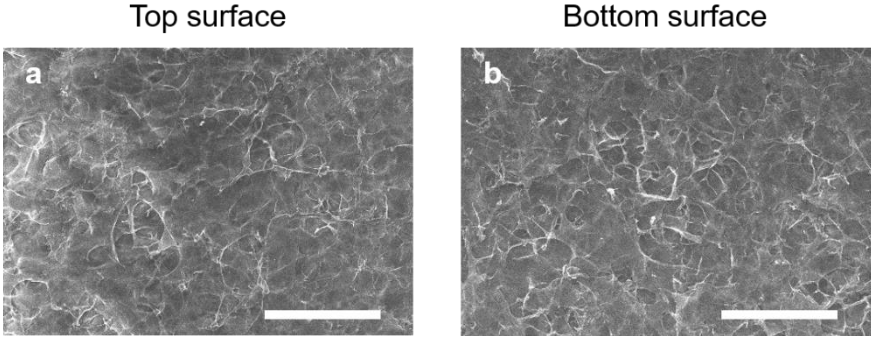

Supplementary Figure 2. Surface mophology of heterogenous graphene oxide. SEM images of top (a) and bottom (b) surface of heterogenous graphene oxide. Scale bars: 50 μm.

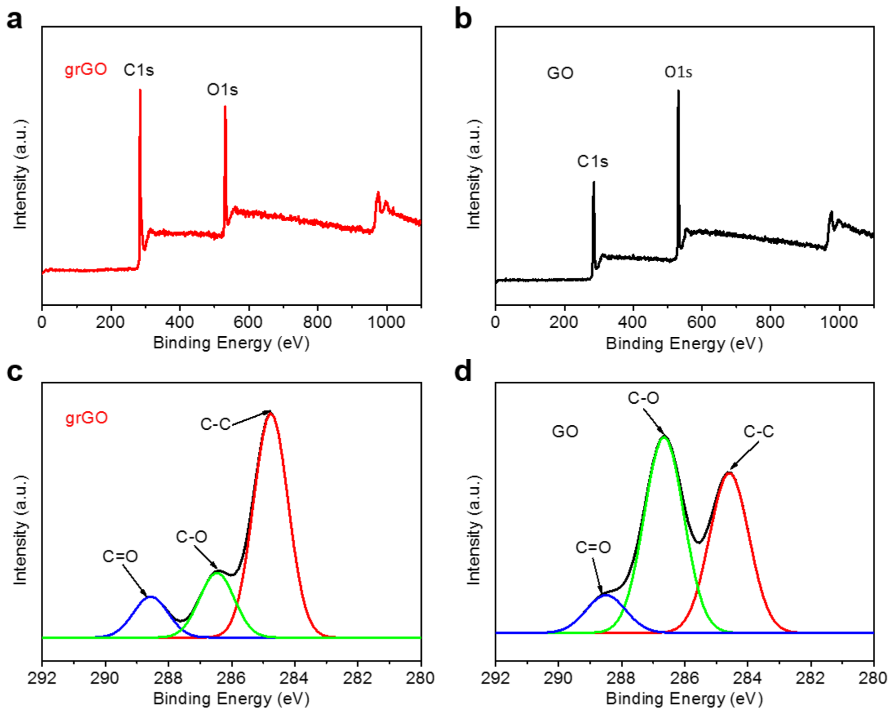

Supplementary Figure 3. Chemical composition analysis of heterogenous graphene oxide. (a, b) XPS spectra of heterogenous graphene oxide (h-GO) on gradient reduced graphene oxide (grGO) and graphene oxide (GO) surface, respectively. (c, d) The high-resolution XPS spectra of C1s peak of grGO and GO surface of h-GO, respectively. The XPS survey spectra of both the grGO and GO surfaces show predominant graphitic C 1s peak at *ca.* 284 eV and O 1s peak at ca. 532 eV[1, 2]. The C/O atomic ratio for the grGO side is *ca.*3:1, significantly higher than that of GO side (*ca.*2:1), demonstrating the distribution variation of oxygen containing functional groups within the h-GO, and this was consistent with the EDS results (Fig. 2c). Moreover, the high-resolution C 1s spectra confirmed the presence of C–C bands (284.7 eV), C–O bands (286.6 eV) and C=O bands (288.5 eV) on both grGO side and GO side. The grGO side had the weakened C–O and C=O peaks than that of GO side, indicating that the oxygen-containing groups on the top grGO part of h-GO were partially removed. These results also explained the inconsistent interlayer spacing change of two sides of h-GO observed by XRD as shown in Fig. 1d.

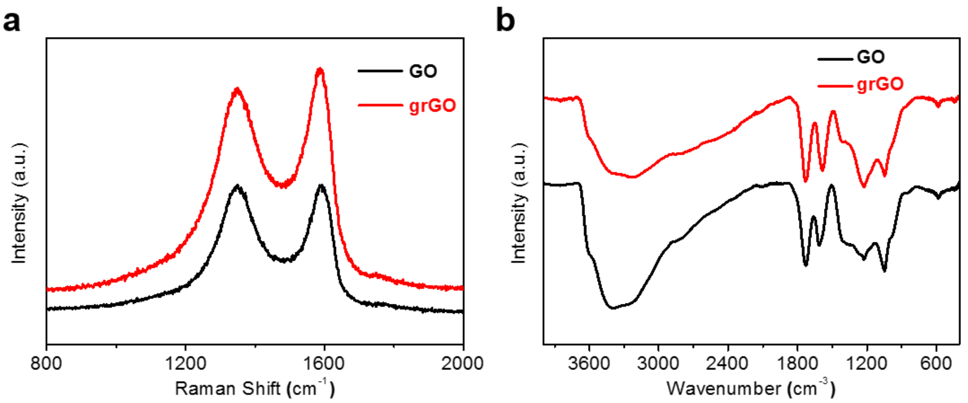

Supplementary Figure 4. Chemical spectral analysis of heterogenous graphene oxide. (a, b) Raman and IR spectrum of gradient reduced graphene oxide (grGO) surface and graphene oxide (GO) surface on heterogenous graphene oxide (h-GO). The Raman spectrum of both grGO and GO showed apparent disorder D band at 1350 cm^{-1} and graphitic G band at 1587 cm^{-1} [2, 3]. The calculated I_D/I_G ratio for the grGO side was 0.92, slightly lower than that of GO side (*ca.*1), suggesting partially removal of OCGs defects within the grGO side. Moreover, FT-IR spectrum of both grGO and GO confirmed the appearance of abundant oxygen-containing functional groups. The broaden and intensive peak at 3380 cm^{-1} revealed O–H bonding. The obvious peak centered at 1730 cm^{-1} was attributed to the vibration of C=O bonding. The sharp peak at 1585 cm^{-1} was associated with the vibration of aromatic C=C stretching. The absorption peak appeared at 1418 cm^{-1} was caused by the deformation vibration of O–H bonding. The peak emerged at 1224 cm^{-1} was consistent with the C–OH stretching. The characteristic peak at 1050 cm^{-1} was assigned to the stretching of C–O bonding[1, 4].

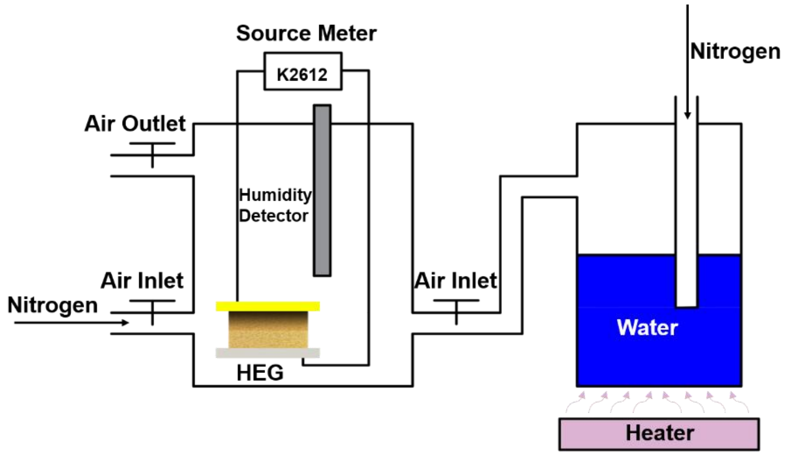

Supplementary Figure 5. Schematic illustration of the relative humidity controlling system. Experimental set-up of the relative humidity (RH) controlling system for measuring the electric output of HEGs. Typically, we simply immobilized the flow rate of the dry nitrogen at a constant speed (1.4 m s^{-1}), tuning the flow rate of the wet nitrogen with different speed to get the variable humidity variation.

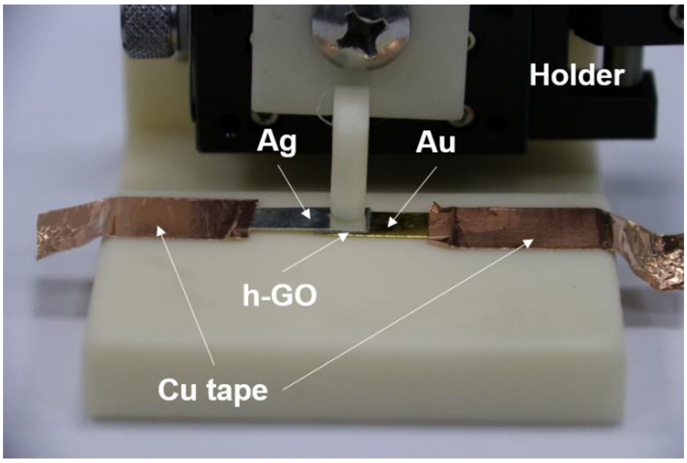

Supplementary Figure 6. Photo of as-fabricated HEG (Ag/GO-grGO/Au).

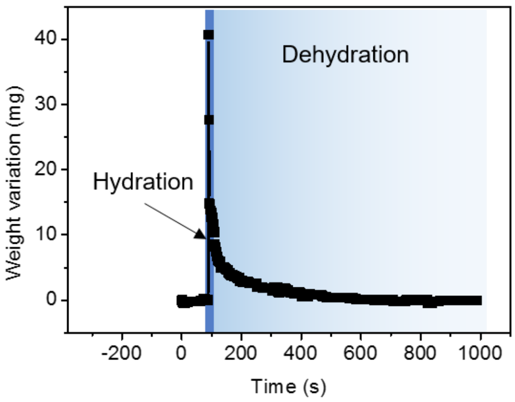

Supplementary Figure 7. Hydration-dehydration process of heterogenous graphene oxide.

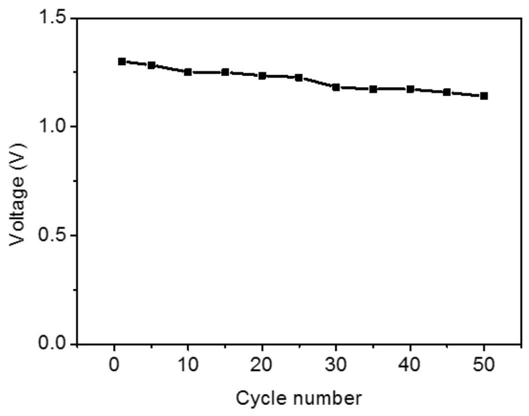

Supplementary Figure 8. Cycling stability of HEG (Ag/GO-grGO/Au).

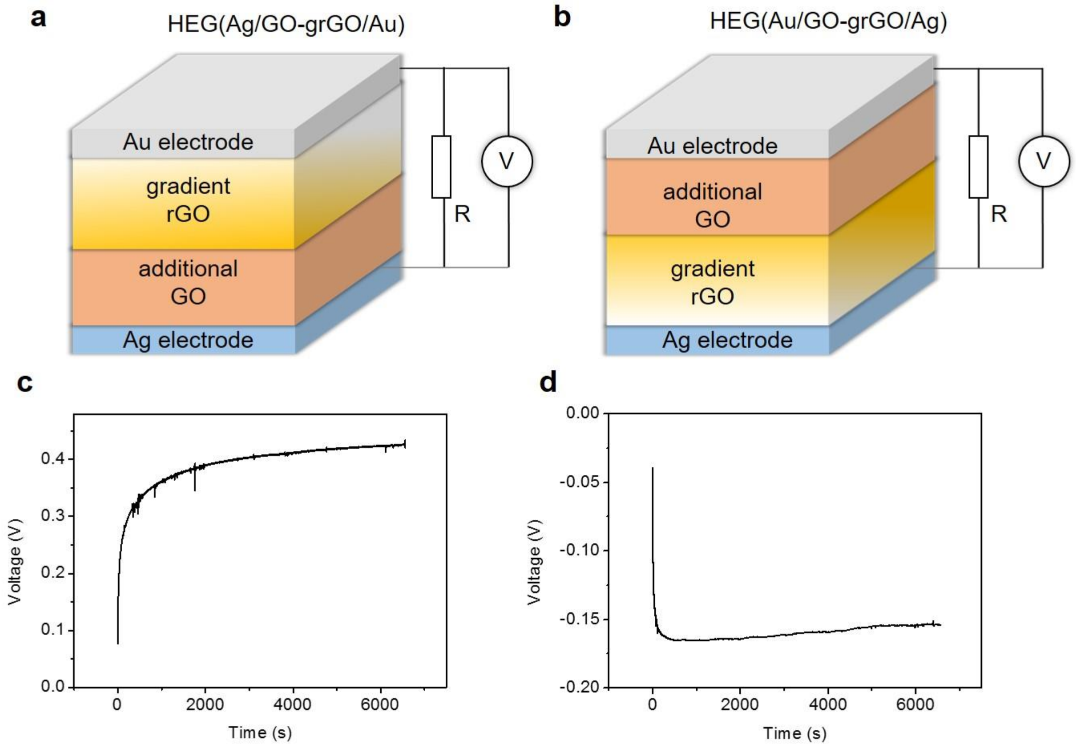

Supplementary Figure 9. Interface influence on hygroelectric generator performance. (a, b) Illustration of device structure of HEG (Ag/GO-grGO/Au) and HEG (Ag/grGO-GO/Au). (c, d) The corresponding generated voltage at its initial state under a constant RH (~15%).

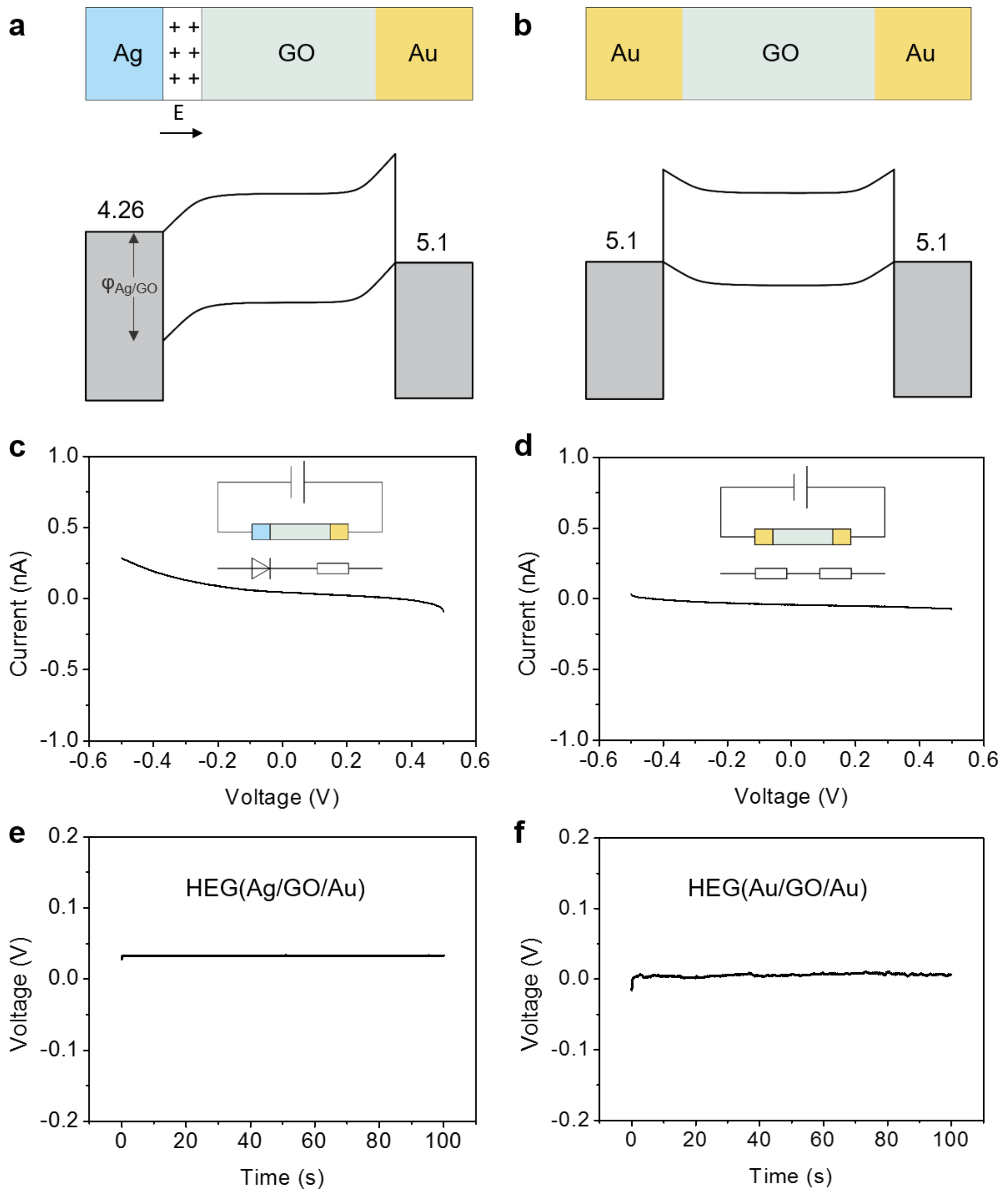

Supplementary Figure 10. Comparation of homogenous graphene oxide based hygroelectric generators. (a–d) Device structure, energy band diagram and corresponding *I*–*V* characteristic curve of (a) HEG (Ag/GO/Au) and (b) HEG (Au/GO/Au). *I*–*V* curves show Schottky diode characteristic of HEG (Ag/GO/Au) and Ohmic contact of HEG (Au/GO/Au). The inset of *I-V* curve is the corresponding test circuit and equivalent circuit diagram. (e, f) Baseline voltage of HEGs with different electrodes configuration.

Supplementary Note 1.

The significant ~0.4 V baseline voltage of HEG (Ag/GO-grGO/Au) arises in the electricity generation process at initial state (RH~15%) (Supplementary Fig. 9c), which should be ascribed to the synergistic effect of inherent asymmetrical chemical structure of h-GO and well-designed interface system of device. As we have proved above, h-GO has compact and intact structure with grGO and homogeneous GO parts. In the grGO section of h-GO, free protons induced by its favorable water absorption ability would unidirectional diffuse from high to low oxygen groups side even at initial low RH in environment. While GO section in h-GO behaves as a protons reservoir that contains a number of movable but genteelly releasing protons because of its homogeneous chemical compositions. Free protons within h-GO induced by the hydration between water molecules and oxygen containing functional groups would reach an equilibrium state, whereby an inner built-in potential establishes. Nevertheless, free electrons can't flow to balance this electric potential as a result of the infinite resistance (1000 MΩ) of external circuit. Therefore, the HEG delivers a sustained baseline voltage.

When changing the polarity of h-GO to construct HEG (Ag/grGO-GO/Au), the generated voltage is distinctly decreased to ~0.16 V (Supplementary Fig. 9d). Compared with HEG (Ag/GO-grGO/Au), the decline of baseline voltage in HEG (Ag/grGO-GO/Au) is attributed to the lack of space charge zone at the Ag and grGO interface, indicating the significant impact of device interface system. Moreover, after switching the polarity of h-GO, the electric output signal is reversed, further confirming that the baseline voltage is inherently derived from the HEG rather than a fake signal.

To clarify the role of asymmetrical chemical structure in the baseline, the h-GO was replaced by homogenous GO with uniform chemical structure as hygroscopic materials in HEG and other conditions kept unchanged, whereby a rather small electric potential of ~0.03 V (Supplementary Fig. 10e) occurred, which should be ascribed to the symmetrical contact potential at two electrode/GO interface. However, by fabrication HEG (Au/GO/Au) with homogenous GO and two identical Au electrodes, there is little indication of baseline voltage (Supplementary Fig. 10f), indicating the significant effect of asymmetrical chemical structure on the origin of baseline voltage.

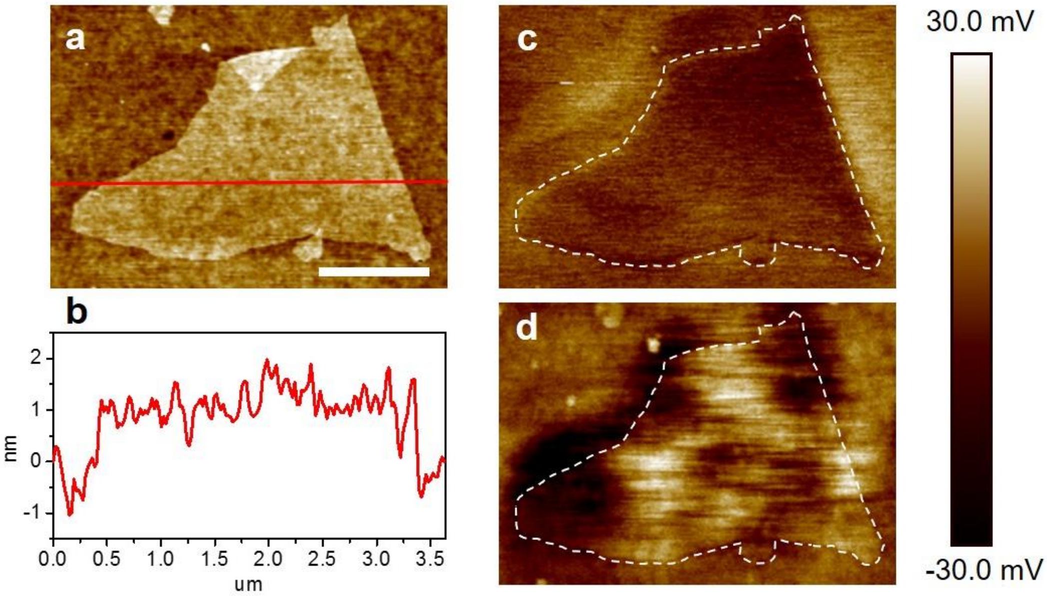

Supplementary Figure 11. AFM analysis of graphene oxide and reduced graphene oxide nanoflakes. (a, b) AFM image and corresponding height profile of a single GO sheet on a mica substrate. Scar bar: 1 μm. (c, d) EFM images of a single GO sheet and an in-situ laser-induced rGO sheet at high RH environment (~80%). The EFM-phase images showed apparent color difference of GO and laser-induced rGO, which are consistent with the dielectric constant difference of them at given RH atmosphere[5]. According to the capacitor model, the phase variation of EFM image corresponds to surface potential[6], which reveals the charge amount of materials on the surface. Consequently, the phase variation reveals the charge difference of GO and laser-induced rGO.

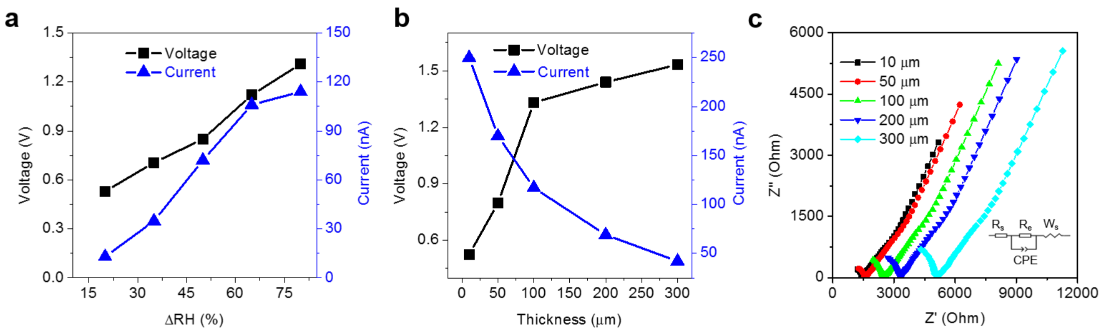

Supplementary Figure 12. The electric performance of hygroelectric generator upon various relative humidity variation and thickness of heterogenous graphene oxide. (a) Voltage, current output in response to the different relative humidity variations (△RH) of HEG (Ag/GO-grGO/Au) with an h-GO thickness of 100 um. (b) Voltage, current output in response to △RH = 80% of HEG (Ag/GO-grGO/Au) with different h-GO thicknesses. (c) The electrochemical impedance spectra of h-GO with different thickness (RH~ 90%, 25 ^{o}C).

Supplementary Note 2.

The output performance of HEG demonstrates a positive dependency on the RH variation of both voltage and current, which should be attributed to the more ionized protons and remarkable charges separation with incremental RH change. As shown in Supplementary Fig. 12b, the voltage output is also enhanced with the increase in thickness of h-GO, while the current output is weakened accordingly. As demonstrated in equation (4) in main text, the generated voltage (*V*) of HEG mainly depends on the charges partition of positive-negative ions pair within h-GO, where d_1 and d_2 represent the thickness of grGO layer and GO layer within h-GO, respectively. This means the generated voltage (*V*) is proportional to the thickness of h-GO (d_1 *and* d_2). Therefore, the generated voltage is enhanced with incremental thickness of h-GO. However, the increased thickness of h-GO could prolong the water absorption time and increase the

migration path for protons that can gradually reduce the ionic conductivity of h-GO, which should be the reason that weakens the output current value of HEG.

To further confirm the ionic conductivity change of h-GO with increased thickness, the electrochemical impedance spectroscopy measurements are implemented on the h-GO with different thickness. For the ionic conductivity measurement, the impedance spectra obtained yielded a depressed semicircle with a slanted line at lower frequencies as shown in Supplementary Fig. 12c. In solid electrolyte system, the corresponding equivalent circuit for this type of spectra is typically represented by electrode resistance (R_s) in series with a parallel combination of electrolyte resistance (R_e) and capacitance[7, 8]. Therefore, the impedance data at high frequencies are fitted by Z-view based on the equivalent circuit (inset Supplementary Fig. 12c), where the depressed semicircles are simulated by the electrolyte resistance in parallel with a Constant Phase Element (CPE) that is generally a result of electrode roughness. The experimental results show that the ionic resistance is actually increased with the thickness of h-GO, indicating a poor ionic conductivity for thicker samples. Therefore, the induced current is weakened whereas the output voltage is promoted with an increase in thickness of h-GO.

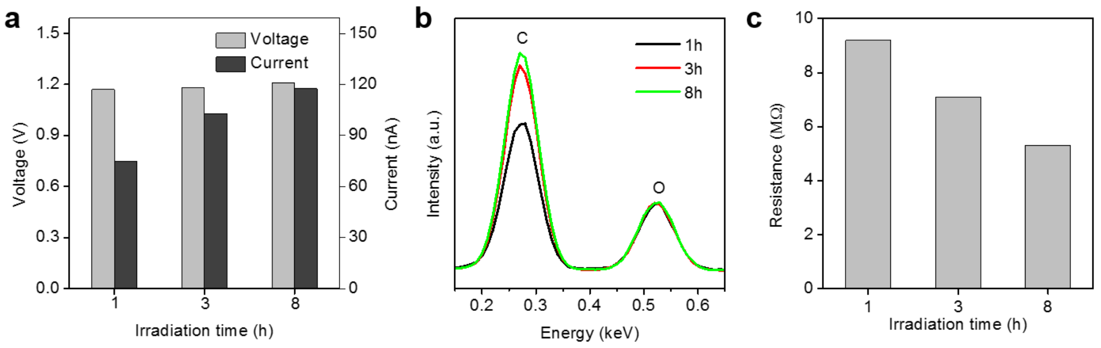

Supplementary Figure 13. The influence of reduction degree on the hygroelectric generator performace. (a) Voltage, current output in response to the △RH = 80% of HEG (Ag/GO-grGO/Au) with different laser irradiation time. (b) EDS spectrum of grGO side within the h-GO with various irradiation time normalized using the oxygen peak. (c) Electrical resistance of h-GO with various irradiation time.

Supplementary Note 3.

By controlling the laser irradiation time for directionally-reduction of h-GO from 1 h to 8 h, the induced voltage is almost unchanged while the current is observably increased. According to equation (4) in main text, the generated voltage (V) is proportional to the difference of charge quantity of (Q_2-Q_1) and (Q_3-Q_2). The reduced oxygen containing functional groups on h-GO along with increased laser irradiation time would induce the electrical resistance decrease of h-GO (from 9.2 MΩ to 5.3 MΩ), thus resulting depletion in numbers of positively charged protons and changes of Q_1, Q_2 and Q_3. However, this concurrent reduction on Q_1, Q_2 and Q_3 would bring insignificant change on the difference of charge quantity (Q_2-Q_1) and (Q_3-Q_2). Therefore, the generated voltage of HEG is nearly unchanged upon variation of electrical resistance of h-GO. However, the positively charged protons diffusion is severely hindered by the increased electrical resistance of h-GO, leading to observably weakened current output of HEG.

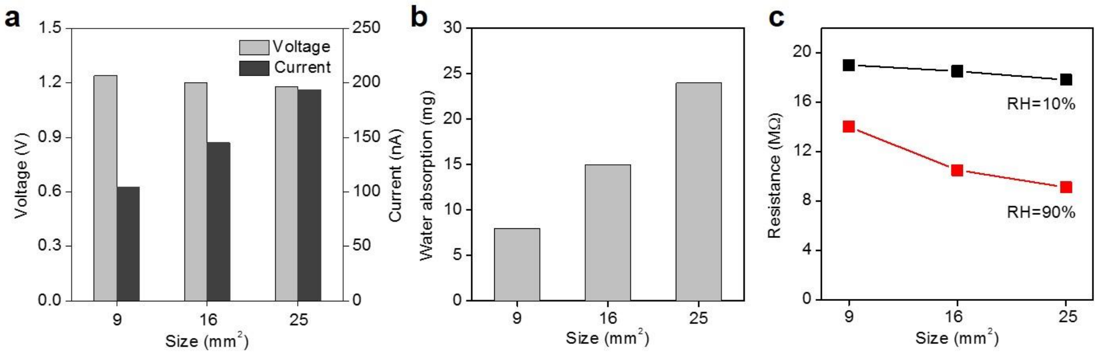

Supplementary Figure 14. Analysis of the size effect. (a) Voltage, current output in response to the △RH = 80% of HEG (Ag/GO-grGO/Au) with different sample size. (b) The water absorption characteristic of samples with different area size. (c) Resistance variation of samples with different size under specific humidity.

Supplementary Note 4.

The electricity generation of HEGs presents a positive dependency between the area size and the output current, coupled with a slightly weakened output voltage with increased area size. This phenomenon could be ascribed to the enhancement effect of improved hydration based on area size increase[8]. As shown in Supplementary Fig. 14b, the water uptake ability of h-GO is remarkably enhanced with incremental area size, which causes more ionized protons and facilitates its migration within h-GO, giving rise to enhanced output current. The slightly shrinking output voltage with increased area size of h-GO could be attributed to the uneven absorption of water molecules due to the incremental sample size. In addition, the electrical resistance of h-GO is reduced with increased thickness.

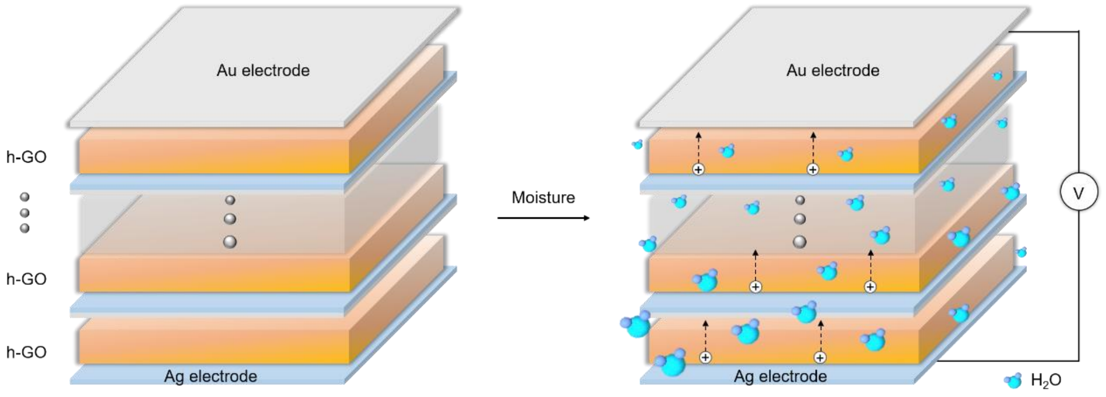

Supplementary Figure 15. Schematic illustration of hygroelectric generator integration with concurrent moisture stimuli and synchronously protons migration. Pertaining to the electricity generation process of the developed HEG in present work, the performance of HEG is significantly relied on the variation of RH. Considering the scaling process, the concurrent stimuli of each unit plays an important role for devices integration. In this way, the consistency of RH change for many devices is vital. Benefited from the sandwiched structure of HEG, the margins of inner h-GO could be fully exposed to ambient environment. Once exposed to moisture, hygroscopic h-GO of each unit can easily adsorb water molecules from environment at the same time[9]. Therefore, this makes sure the synchronously charges separation and protons migration within different units, thus leading to the consistency of electricity generation.

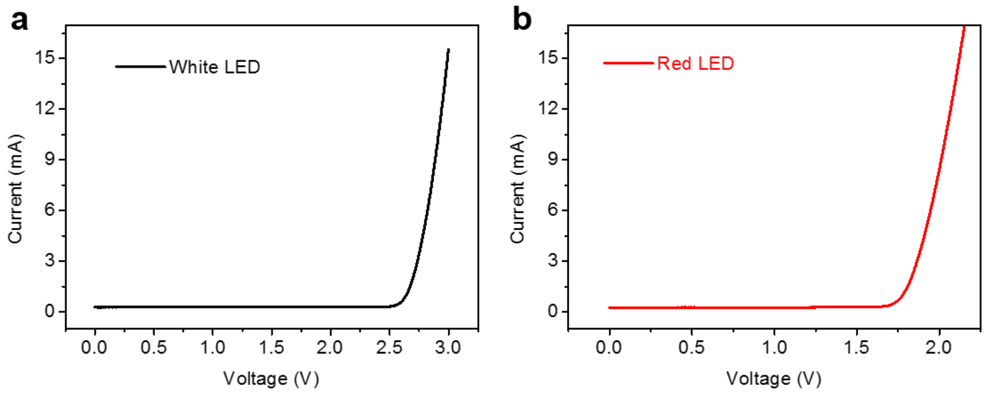

Supplementary Figure 16. Current-voltage curves of white and red light-emitting diodes.

Supplementary references

1. Xu, Y. X., Bai, H., Lu, G. W., Li, C., & Shi, G. Q. Flexible graphene films via the filtration of water-soluble noncovalent functionalized graphene sheets. *J. Am. Chem. Soc.* **130**, 5856–5857 (2008).
2. Yang, D., *et al.* Chemical analysis of graphene oxide films after heat and chemical treatments by X-ray photoelectron and Micro-Raman spectroscopy. *Carbon* **47**, 145–152 (2009).
3. Malard, L. M., Pimenta, M. A., Dresselhaus, G., & Dresselhaus, M. S. Raman spectroscopy in graphene. *Phys. Rep.* **473**, 51–87 (2009).
4. Dreyer, D. R., Park, S., Bielawski, C. W., Ruoff, R. S. The chemistry of graphene oxide. *Chem. Soc. Rev.* **39**, 228–240 (2010).
5. Moser, J., Verdaguer, A., Jimenez, D., Barreiro, A., & Bachtold, A. The environment of graphene probed by electrostatic force microscopy. *Appl. Phys. Lett.* **92**, 3 (2008).
6. Kulkarni, D. D., Kim, S., Chyasnavichyus, M., Hu, K. S., Fedorov, A. G., & Tsukruk, V. V. Chemical reduction of individual graphene oxide sheets as revealed by electrostatic force microscopy. *J. Am. Chem. Soc.* **136**, 6546–6549 (2014).

7. Gao, W., et al. Direct laser writing of micro-supercapacitors on hydrated graphite oxide films. *Nat. Nanotechnol.* **6**, 496-500 (2011).

8. Zhao, F., Liang, Y., Cheng, H. H., Jiang, L., & Qu, L. T. Highly efficient moisture-enabled electricity generation from graphene oxide frameworks. *Energy Environ. Sci.* **9**, 912-916 (2016).

9. Bi, H. C., et al. Ultrahigh humidity sensitivity of graphene oxide. *Sci. Rep.* **3**, 7 (2013).
