# Supplementary material for: Interface-mediated hygroelectric generator with an output voltage approaching 1.5 volts
Source: Nat Commun. 2018 Oct 9;9:4166. doi: 10.1038/s41467-018-06633-z (PMC6177432; doi:10.1038/s41467-018-06633-z)
Supplement: Supplementary file 3 — Description of Additional Supplementary Files [file 41467_2018_6633_MOESM3_ESM.pdf]

Description of Additional Supplementary Files

File Name: Supplementary Movie 1

Description: The demonstration of hygroelectric generator package as a practical power source for feeding commercial electronics. The “THU” pattern consisted of 19 commercial LEDs in series is lighted by periodically switching on the designed power-supplying system.
